# Gender of children and social provisions as predictors of unplanned pregnancies in Pakistan: a cross-sectional survey

**DOI:** 10.1186/s13104-018-3696-8

**Published:** 2018-08-14

**Authors:** Sadiq Naveed, Usman Ghani Lashari, Ahmed Waqas, Mariam Bhuiyan, Hafsa Meraj

**Affiliations:** 1 KVC Prairie Ridge Hospital, 4300 Brenner Drive, Kansas, KS 66104 USA; 2 United Arab Emirates University, Al-Ain, Abu Dhabi United Arab Emirates; 3 CMH Lahore Medical College & Institute of Dentistry, Lahore Cantt, Pakistan; 4 Human Development Research Foundation, Rawalpindi, Pakistan; 5 Johns Hopkins University School of Medicine, Baltimore, MD USA; 6 Sharif Medical and Dental College, Jati Umra, Lahore, Pakistan

**Keywords:** Social support, Pregnancy, Family planning, Pakistan, Unwanted pregnancies, Motherhood, South Asia

## Abstract

**Objective:**

Previous research indicates that attitudes to pregnancy and motherhood are influenced by social values, culture and religion. This study explores the relationship between social support and unwanted pregnancy among Pakistani women. This cross-sectional study was conducted at four teaching hospitals in Lahore in 2014.

**Results:**

A total of 500 pregnant women who visited the hospitals’ obstetrics and gynecology departments were asked to respond to a questionnaire consisting of respondents’ characteristics and the Social Provisions Scale (SPS). Logistic regression analyzed the predictors of unplanned pregnancy. Unwanted pregnancies were more likely to occur among pregnant women from rural areas, with low scores on the SPS ‘reassurance of worth’ sub-scale, no history of contraceptive use, and who already had at least one son than those with no sons.

**Electronic supplementary material:**

The online version of this article (10.1186/s13104-018-3696-8) contains supplementary material, which is available to authorized users.

## Introduction

Unplanned pregnancies are defined as pregnancies that occur at inconvenient times in women’s lives or pregnancies that are unwanted. They tend to cause hardship for families, and jeopardize the mental and physical health of millions of women and children worldwide. Problems include: late initiation of prenatal care, low birth-weight, child abuse and neglect, behavioral problems in children, lack of folic acid supplementation, low hemoglobin levels, and negative labor experience [1]. A close association has been found with antenatal depression and anxiety [2]. All of these factors increase neonatal care costs, and other costs related to long-term disability in women and babies [3]. Moreover, a frequent outcome of unintended pregnancy is induced abortion. In countries such as Pakistan where it is illegal, women resort to untrained midwives and untrained health professionals [4].

A number of studies have reported risk factors associated with unwanted pregnancies in different regions and cultures. They include socioeconomic disparities, lack of social support, the mother’s age, parity and maternal behaviors such as smoking and alcohol consumption [5, 6]. These risk factors are stable across cultures and found in different regions of the world. However, several culture-specific predictors of unwanted pregnancies have been reported in the literature. For instance, Hussain et al. found that the gender of older offspring is a major predictor of unwanted pregnancies in Pakistan [7]. Interestingly, in Southeast Asia, this factor also seems to play a role in guiding attitudes to unwanted pregnancies. Daughters are seen as inferior to sons, and consequently have poor access to healthcare and educational opportunities [2, 8]. This sociocultural difference contributes to honor killings, the bride price, and dowry system, the lower status of female legal testimony, forced marriages, and the denial of a woman’s right to have a career [2, 8]. In contrast, sons are viewed as breadwinners and agents of the continuation of the family name.

Social support encompasses guidance and advice, help, care and self-esteem. It not only benefits the physical and mental health of the general populace, but also helps pregnant women to make informed decisions [9, 10]. Perceived social support has also been shown to be a strong predictor of attitudes, practices and knowledge regarding unwanted pregnancies. In a similar context, studies have shown that social support plays a significant role in preventing the negative health consequences of pregnancy [2]. Support from the family, and society in general, helps a woman adjust to her new social role as a mother [11]. This becomes even more important in a patriarchal setting like Pakistan where information regarding wanted/timed pregnancy is scarce, and attitudes to contraception are negative.

Unfortunately, there is a dearth of data exploring the factors associated with unintended pregnancies in Pakistan, thus, warranting this study. This study explores the factors associated with unintended pregnancies, with a focus on gender disparity, family systems, and societal support among Pakistani women.

## Main text

### Methods

This cross-sectional study was conducted in the obstetrics and gynecology departments of four teaching hospitals at Lahore, Pakistan, from February 2014 to June 2014. These hospitals are state-funded, and serve lower- or lower-middle-income populations in surrounding areas. All pregnant women assessed as having low or lower middle income, who visited the departments for routine antenatal care were included. Women with major medical comorbidities and receiving inpatient treatment were excluded from current study. All participants gave written informed consent, and approval for the study was granted by the Research Ethics Review Committee of CMH Lahore Medical College, and the Institute of Dentistry, Lahore, Pakistan.

Data was collected conveniently by four, final-year medical students, using a pre-prepared questionnaire. Prior to data collection phase, these students received a 2-day interviewing and empathy skills workshop by trained psychologists, also ensuring a good inter-rate reliability. Participants provided demographic information, family structure and were asked to evaluate the state of their pregnancy, Hospital Anxiety and Depression Scale (HADS) and perceived levels of social support using the Social Provisions Scale (SPS). More details about the study design can be read elsewhere [2].

Perceived social support was assessed with Urdu version of the SPS [12], originally developed by Weiss [13]. This scale has been cross-culturally validated among the Pakistani population, and has been shown to have excellent psychometric properties [12]. Internal consistency was very high in the present study sample (Cronbach’s alpha = 0.92). It consists of 24 questions describing different characteristics of participants’ social support system. It assesses responses on a 4-point response; 1 (strongly disagree) to 4 (strongly agree).

According to Weiss [13], social support is a multi-dimensional concept consisting of six “provisions”, which may be obtained from relationships with others [13]. It is generally believed that individuals require all of them to feel adequately supported [14]. The six provisions as assessed with SPS are: (a) attachment: emotional closeness from which one derives a sense of security; (b) social integration: a sense of belonging to a group of people who share common interests, recreational activities and concerns; (c) reassurance of worth: acknowledgment of one’s competence and skills; (d) reliable alliance: the assurance that one can count on others for assistance under any circumstances; (e) guidance: advice and information; and (f) opportunities to nurture: the sense that others rely upon one for their well-being [13, 14].

Post-hoc power analysis with GPower (v 3.1.7) revealed that a power of 84% was achieved using following parameters: sample size of 500, alpha (0.05), R^2^ with other covariates (0.04), probability of unplanned pregnancies with high social support (30%) and low social support levels (43%).

All data were analyzed in SPSS (version 20). Categorical data were presented as frequencies, and quantitative data as means (SD). Association between parity among mothers and status of pregnancy was explored using point-biserial correlation. Partial correlation was used to assess association between SPS scores and status of pregnancy, after controlling for age of the respondents. Logistic regression analyzed predictors of unplanned pregnancy. Education, background (rural vs urban), household income (low vs high), number of daughters and sons, and scores on Social Provisions Scale were entered as covariates in the regression model. Prior to running the regression analysis, Box–Tidwell (1962) test was used to ascertain the linearity of the continuous variables with respect to the logit of the dependent variable. An alpha value < 0.05 was considered significant.

### Results

The final sample consisted of 500 respondents. The mean age was 27.41 (5.65), most were from rural areas, from a low socioeconomic class, and had not completed high school. The majority reported that their current pregnancy was unplanned (365, 73%). A total of 276 (55.2%) of women had a prior history of contraceptive use. An analysis of HADS scores showed that a high percentage of women suffered from antenatal anxiety (245, 49%) and depression (159, 31.8%). The demographic characteristics of respondents and their mean (SD) SPS scores are presented in Table 1. Our findings related to antenatal anxiety and depression in this sample can be read elsewhere [2] (Additional file 1).

**Table 1:** Demographic characteristics and SPS scores for pregnant women in Lahore (n = 500)

Variable	Frequency (n)	Percentage (%)	Mean (SD)
Pregnancy			
Planned	135	27	
Unplanned	365	73	
Education			
Illiterate	85	17	
Literate	415	83	
Background			
Rural	182	36.4	
Urban	318	63.6	
Monthly household income			
≤ 81.11 USD	148	29.6	
> 81.11 USD	352	70.4	
Having daughters			
Yes	236	47.2	
No	264	52.8	
Having sons			
Yes	278	55.6	
No	222	44.4	
Used family planning previously			
Yes	224	44.8	
No	276	55.2	
Parity			
Primipara	160	32.0	
1 child	122	24.4	
2 children	90	18.0	
≥ 3 children	128	25.6	
Social Provisions Scale			72.32 (12.20)
Attachment			11.83 (2.43)
Social integration			11.85 (2.77)
Reassurance of worth			11.95 (2.69)
Reliable alliance			12.48 (2.39)
Guidance			12.29 (2.41)
Nurturance			11.91 (2.46)

Parity among mothers was associated positively with unplanned pregnancies (r = 0.19, *p* < 0.001) and insignificantly with social support (r = − 0.04, *p* > 0.05). The partial correlations showed that unplanned pregnancy was negatively associated with the total score on the SPS scale (r = − 0.19, *p* < 0.001), which persisted even after controlling for the age of the respondent (r = − 0.19, *p* < 0.001).

The logistic regression model was statistically significant (*p* < 0.001). Hosmer and Lemeshow Chi square statistics was non-significant (*p* = 0.335, χ^2^ = 9.08). Logistic regression predicted 75.6% of the model correctly, unplanned pregnancies 93.4%, planned pregnancies (27.4%), and explaining 21.1% of variance in unwanted pregnancies. Women from rural areas had higher odds of having an unplanned pregnancy than their urban counterparts. Low scores on both reassurance and nurturance subscale exhibited an increased likelihood of unplanned pregnancy among the participants. The history of use of contraceptives halved the likelihood of unplanned pregnancies, however, those pregnant women who had one or more sons exhibited higher odds of unplanned pregnancies than their counterparts. On the other hand, education level, household income, having daughters, total number of children, attachment, social integration, reliable alliance, and guidance were insignificant predictors (Table 2).

**Table 2:** Predictors of unplanned pregnancy in pregnant females (n = 500)

Variables	Subcategories	B	Standard error in B	*p*	Odds ratio (OR)	95% CI for OR	
						Lower	Upper
Education	Literate	− 0.295	0.303	0.330	1		
	Illiterate				0.744	0.411	1.348
Background	Rural	0.968	0.276	< 0.001	1		
	Urban/semi-urban				2.633	1.533	4.522
Household income	Low	0.264	0.266	0.322	1		
	High				1.302	0.772	2.194
Having daughters	Yes	− 0.033	0.287	0.908	1		
	No				0.968	0.551	1.698
Having sons	Yes	0.701	0.292	0.016	1		
	No				2.02	1.137	3.571
Total children	≤1	− 0.415	0.362	0.251	1		
	≤ 2				0.660	0.325	1.341
Used contraceptives	No	0.497	0.249	0.046	1		
	Yes				1.644	1.009	2.678
Attachment		0.117	0.080	0.146	1.124	0.960	1.315
Social integration		− 0.074	0.054	0.173	0.929	0.835	1.033
Reassurance of worth		0.133	0.056	0.018	1.143	1.023	1.276
Reliable alliance		− 0.017	0.076	0.819	0.983	0.846	1.141
Guidance		0.050	0.085	0.556	1.051	0.890	1.242
Nurturance		− 0.101	0.060	0.089	0.904	0.804	1.016
Constant		− 2.397	0.812	0.003	0.091		

Cox and Snell R^2^ = 14.5%, Negelkerke R^2^ = 21.1%

### Discussion

Our study found a high prevalence of unwanted pregnancies among Pakistani women who lived in rural areas, scored low on the ‘reassurance of worth’ SPS subscale, had no history of using contraceptives, and already had sons. Our findings are comparable with statistics reported in Karachi (38%) and other developing and third-world countries such as Brazil (n = 55.8%), but are much higher than the developed world, where estimates are comparatively low—for example 27% in Canada [4, 15, 16].

Our study showed that unplanned pregnancies are consistent with a lack of social support, particularly the ‘reassurance of worth’ SPS subscale. Our findings are in concordance with Shahry et al. who reported that women with unwanted pregnancies exhibit low social support and self-efficacy [17]. Social support is an important buffer in stressful situations, such as during an unintended pregnancy, the lack of which may lead to a variety of psychopathologies such as perinatal anxiety and depression and deleterious health consequences for the fetus [2, 9, 13]. These trends have also been demonstrated among British women with unplanned pregnancies who reported lower levels of perceived support and less contact with friends and family, leading to feelings of isolation and negative attitudes toward their pregnancy [18].

Reassurance of worth is one of the six provisions described by Weiss, and refers to an acknowledgment of one’s competence and skills, and an admiration of one’s talents and abilities [13]. It is interesting to note that, according to Weis, reassurance of worth is often provided by co-workers [13]. This is particularly relevant in the Pakistan socio-cultural setting where, in some traditional families, working women are stigmatized. The home is considered the appropriate place for women, and their role is to be an obedient wife and a loving mother. Several studies have found that Muslim women tend to place a very high value on motherhood, more so than women from other religious and cultural backgrounds [19, 20]. Therefore, in the context of Pakistan, reassurance of worth may also be derived from the home environment.

According to Grav et al. emotional support has been found to be particularly important for women [9]. This explains why women with a lack of social support tend to exhibit adverse emotional symptoms. In eastern societies, there is a strong cultural taboo against pregnancies in certain groups, notably unmarried women. Such pregnancies are likely to be unplanned, there is very little social support, and the mothers are castigated by the community, leading to a lack of reassurance of worth public and internalized stigmatization and social discrimination [21]. Affirming the role of women as mothers and wives, and empowering them to make their own decisions may lead to a lower prevalence of unplanned pregnancies.

Our analysis revealed that women with a history of using contraceptives were less likely to have an unplanned pregnancy. According to the Demographic and Health Survey conducted by The United Nations Children’s Emergency Fund (UNICEF) in 2015, Pakistan has the second-lowest rate of contraceptive use among South Asian countries, after Afghanistan [22]. In Muslim cultures, motherhood is considered a major milestone, and the fulfillment of a woman’s destiny [23, 24]. Hence it is unsurprising that women are hesitant to use contraception. There is a need to increase awareness of contraceptive use, and improve knowledge of the different methods of contraception, which should be coupled with giving women greater autonomy to make decisions in this area [25].

Our analysis found that women from rural areas were more likely to have unplanned pregnancies than their urban counterparts. This may be because gender discrimination is more evident in rural communities, where women have less autonomy and are more likely to succumb to superstition and traditional values regarding pregnancy [26]. Additionally, our analysis revealed that urban women have higher rates of contraceptive use than those living in rural areas, which also contributes to their lower number of unplanned pregnancies.

Another finding was that unplanned pregnancies are significantly associated with the gender of older children. Mothers who already had sons were more likely to have unplanned pregnancies. Our results are consistent with the statistics reported by Hussain et al. who evaluated the role of the gender of older offspring in reproductive intentions and, thus, the behavior of Pakistani women [7]. This is relevant in the South Asian region, where reproduction is an integral part of patriarchal and heteronormative family systems—sons are seen as the carriers of the family name and breadwinners, and are therefore preferred to their female counterparts. We opine that deterrence to the use of contraceptives because of increasing number of sons among families occurs for a multitude of reasons. One of these reasons include financial security of respective parents in old age, as well as assistance in bringing up the younger offspring. Moreover, elder brothers usually bear the responsibility of assisting parents in collecting dowry for the younger sisters.

However, unlike Hussain et al. our study did not find that already having daughters was significantly associated with status of the pregnancy [7]. This is an encouraging trend, and reflects a sociocultural change that suggests a better attitude toward having daughters. The transition in family systems, women’s agency and fertility has been documented in a number of studies. For instance, Buzdar and Ali, noted a rise in positive attitudes toward educating daughters, despite major disparities in infrastructure and access to schools, among the tribal populace of Dera Ghazi Khan in Punjab, Pakistan [27].

In our study, education was not significantly associated with the number of unplanned pregnancies. It is usual to expect that better education is consistent with a decrease in unplanned pregnancies. However, it is important to note that, unlike more developed countries, most schools in South Asia do not teach reproductive health [28]. In fact, such education is often considered a taboo, and even educated women may lack knowledge [28].

## Limitations

This study has several limitations and therefore, the results should be interpreted with caution. Its cross-sectional nature limits any inferences about causality and temporality between unplanned pregnancy and the associated factors. Convenience sampling and the lack of data for comparison with control groups limits the generalizability of the results. The hospital in which the data was sampled catered mainly to lower- and lower-middle income groups. Therefore, this bias might not reflect the prevalence, attitudes, and factors affecting unplanned pregnancies in the upper socioeconomic classes of Pakistan.

## Additional file

**Additional file 1.** Social provisions, anxiety and depression during pregnancy. This dataset contains all variables pertaining to the publication including demographic characteristics, family composition, social support, anxiety and depression among pregnant women in Pakistan.

## Abbreviations

SPS: Social Provisions Scale
HADS: Hospital Anxiety and Depression Scale
